# A modified frozen elephant trunk technique for acute Stanford type A aortic dissection

**DOI:** 10.1186/s13019-020-01306-9

**Published:** 2020-10-21

**Authors:** Shi-bo Song, Xi-jie Wu, Yong Sun, Shi-hao Cai, Po-yuan Hu, Hai-feng Qiang

**Affiliations:** Department of Cardiovascular Surgery, Xiamen Cardiovascular Hospital, Xiamen university, XiaMen, 361000 China

**Keywords:** Acute Stanford type a aortic dissection, Frozen elephant trunk, Fenestration

## Abstract

**Background:**

Acute Stanford type A aortic dissection is often fatal, with a high mortality rate and requiring emergency intervention. Salvage surgery aims to keep the patient alive by addressing severe aortic regurgitation, tamponade, primary tear, and organ malperfusion and, if possible, prevent the late dissection-related complications in the proximal and downstream aorta. Unfortunately, no optimal standard treatment or technique to treat this disease exists. Total arch replacement with frozen elephant trunk technique plays an important role in treating acute type A aortic dissection. We aim to describe a modified elephant trunk technique and report its short-term outcomes.

**Methods:**

From February 2018 to August 2019, 16 patients diagnosed with acute Stanford type A aortic dissection underwent surgery with the modified frozen elephant trunk technique at Xiamen Heart Center (male/female: 9/7; average age: 56.1 ± 7.6 years). All perioperative variables were recorded and analyzed. We measured the diameters of the ascending aorta, aortic arch, and descending aorta on the bifurcation of the pulmonary and abdominal aortas and compared the diameters at admission, before discharge, and 3 months after discharge.

**Results:**

Fifteen patients (93.8%) had hypertension. The primary tears were located in the lesser curvature of the aortic arch and ascending aorta in 5 (31.3%) and 9 patients (56.3%), respectively, and no entry was found in 2 patients (12.5%). The dissection extended to the iliac artery and distal descending aorta in 14 (87.6%) and 2 patients (12.5%), respectively. The duration of cardiopulmonary bypass (CPB), cross-clamping, and antegrade cerebral perfusion were 215.8 ± 40.5, 140.8 ± 32.3, and 55.1 ± 15.2 min, respectively. Aortic valve repair was performed in 15 patients (93.8%). Bentall procedure was performed in one patient (6.3%). Another patient received coronary artery repair (6.3%). The diameters at all levels were greater on discharge than those on admission, except the aortic arch. After 3 months, the true lumen diameter distal to the frozen elephant trunk increased, indicating false lumen thrombosis and/or aortic remodeling.

**Conclusions:**

The modified frozen elephant trunk technique for acute Stanford type A aortic dissection is safe and feasible and could be used for organ malperfusion. Short-term outcomes are encouraging, but long-term outcomes require further investigation.

## Background

Acute Stanford type A aortic dissection is a lethal event requiring emergency surgery [1]. There is controversy regarding how to treat this disease, especially when the aortic arch and descending aorta are involved (DeBakey type I and II) [2–4]. Early surgeons used a limited approach, such as ascending aortic or hemiarch replacement, which ignored the true lumen flow and pathologic disorders of the descending aorta [5].

Some papers indicate that frozen elephant trunk techniques have an acceptable mortality or morbidity risk for treating acute type A aortic dissection [1, 6–8]. To obliterate the false lumen and better manage the descending aorta for good remodeling, and also to simplify the procedure and avoid time-consuming hemostasis we introduce a new modified technique to achieve the goals mentioned above. The procedure incorporates a frozen elephant trunk that is fenestrated proximal to the stent graft to perfuse the supra-arch vessels. Perioperative data and short-term outcomes are reported.

## Methods

From February 2018 to August 2019, 16 patients presenting with acute Stanford type A aortic dissections were admitted to our hospital. Medical records were retrospectively reviewed. The ethical committee of Xiamen Heart Centre approved for this retrospective study, and all patients provided informed consent. All patients in this study underwent treatment using the modified technique.

All perioperative variables were recorded and analyzed. We measured the diameter of the ascending aorta, aortic arch, and descending aorta at the bifurcation of the pulmonary artery and abdominal aorta and compared the diameter at admission, before discharge, and 3 months after discharge.

### Procedure

**Fig. 1:**
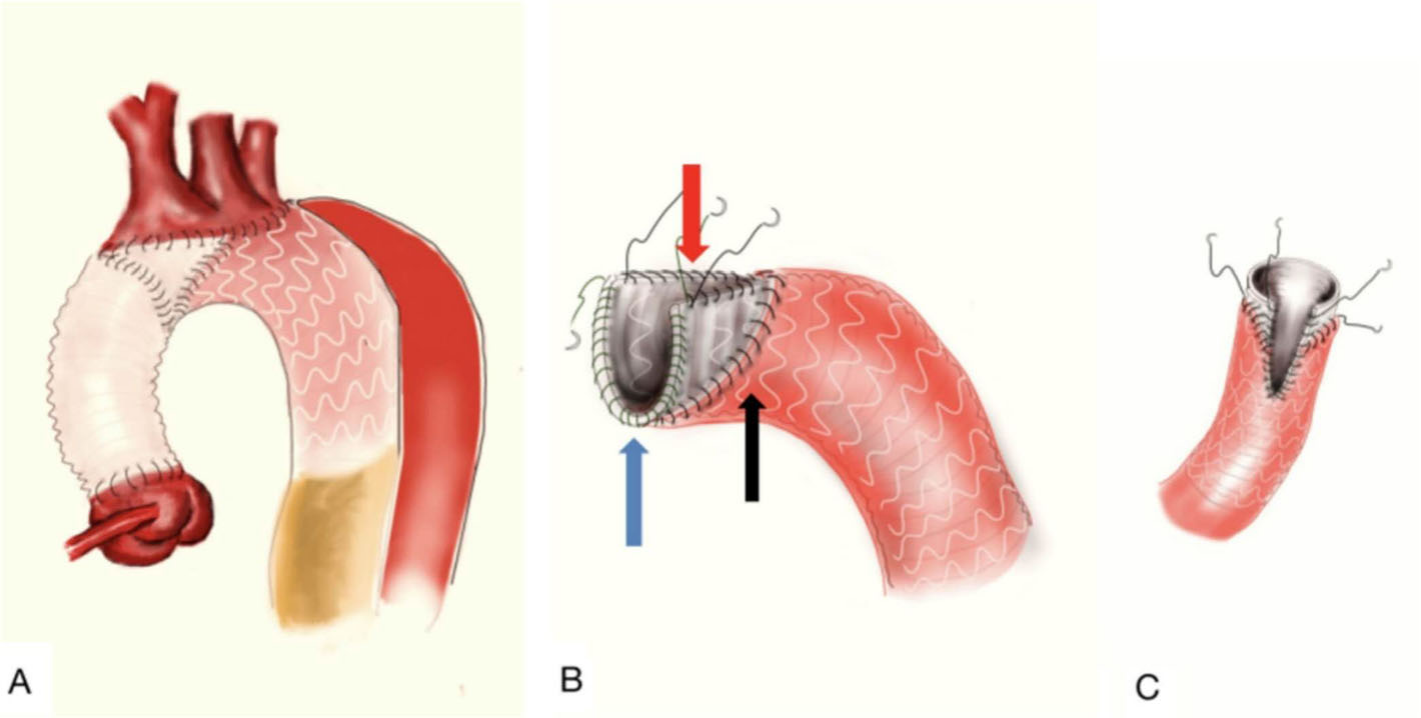
**a**: The illustration of a “fenestrated” frozen elephant trunk for Stanford A dissection; **b**: The red arrow and the black arrow indicate continuous suture and circumferential suture to prevent endoleak; the blue arrow indicates continuous mattress suture. **c**: The illustration of the “fenestration”

After median sternotomy, the three brachiocephalic arteries were dissected carefully and prepared. Cardiopulmonary bypass was then initiated via the femoral artery and right atrium cannulation. The ascending aorta was clamped, the proximal aorta transected, and the aortic valve repaired if necessary. After reconstructing the root with Teflon felt strips, circulatory arrest was started with unilateral cerebral perfusion via innominate artery cannulation [9, 10] at a rate of 5-10 mL/kg/min and rectal temperature of 25 °C. The aortic arch was opened and transected at the innominate artery level to determine whether there was any tear. The diameter of the arch was measured to match an appropriate stent graft size. A 10–15 cm WeiChuang (Cronus; Microport Medical Co, Ltd., Shanghai, China) stent graft was inserted into the true lumen of the arch and released to a level that covered the three brachiocephalic vessels. We partially resected and trimmed the graft as an island fenestration around the three orifices of the brachiocephalic vessels. Then, it was circumferentially sutured to the aortic arch using 4–0 Prolene continuous mattress sutures, just as the root reconstruction method (Fig. 1). Finally, the reinforced fenestrated stent graft-arch stump was anastomosed to the proximal vascular graft. During the arch reconstruction, a silicone Foley catheter with an aqueous capsule was inserted into the stent graft as an endoclamp, which lower body could be perfused simultaneously. Circulatory arrest time could be shortened with this maneuver. The temperature could be rewarmed as well.

### Follow-up

CTA was performed when patients admitted, before discharge, and 3 months after discharge, measuring the diameters of the ascending aorta, true lumen and false lumen of the aortic arch, and the descending aorta. Changes in the diameter of the different sites were recorded and analyzed.

### Statistical analysis

Standard descriptive statistical analyses were used. Continuous variables are shown as the means with standard deviations or as medians with ranges (because of the small sample size), and categoric variables are presented as percentages. Data were analyzed using SPSS version 20.0 (IBM Corporation, Armonk, NY). *P* values < 0.05 were considered significant.

## Results

Fifteen patients (93.8%) had hypertension. The primary tears were located in the lesser curve of the aortic arch in 5 patients (31.3%) and ascending aorta in 9 patients (56.3%), and no entry was found in 2 patients (12.5%). The dissection extended to the iliac artery in 14 patients (87.6%) and to the distal descending aorta in 2 patients (12.5%). The duration of cardiopulmonary bypass, aorta cross-clamping, and antegrade cerebral perfusion were 215.8 ± 40.5, 140.8 ± 32.3, and 55.1 ± 15.2 min, respectively. Intensive care unit time was 9.6 ± 6.1 days. Concomitant procedures included aortic valve repair (15/16, 93.8%), Bentall procedure (1/16, 6.3%), and coronary artery repair (1/16, 6.3%).

### Surgical data

A total of 16 patients who were diagnosed with acute Stanford type A dissection underwent our modified frozen elephant trunk technique. Detailed perioperative variables such as cardiopulmonary bypass time, cross-clamping time, circulatory arrest with cerebral perfusion time, and postoperative variables such as ICU stay and ventilation time are shown in Tables 1 and 2.

**Table 1:** Preoperative clinical characteristics

Characteristic	n(%)
Age	56.1 ± 7.6
Patients	16
Male/female	9/7 (56.3/43.8)
Comorbidities	
Hypertension	15 (93.8)
Chronic kidney disease	1 (6.3)
Diabetes mellitus	3 (18.8)
Primary entry	
Ascending aorta	9 (56.3)
Arch	5 (31.3)
No entry found	2 (12.5)
Malperfusion	
Coronary	1 (6.3)
Leg	2 (12.5)
Cerebral	2 (12.5)

**Table 2:** Intraoperative and postoperative data

Variables	Data
Operative time (min)	376.0 ± 69.5
CPB time (min)	215.8 ± 40.5
Clamp time (min)	140.8 ± 32.3
Antegrade cerebral perfusion time (min)	55.1 ± 15.2
Hospital mortality	0
Postoperative complications	
Intestinal necrosis	1 (6.3%)
Paraplegia	1 (6.3%)
Stroke	1 (6.3%)
AKI requiring dialysis	0 (0)
Postoperative ICU stay(d)	9.6 ± 6.1
Reintubation	2 (12.5%)
Tracheotomy	2 (12.5%)
Concomitant procedures	
Bentall procedure	1 (6.3%)
Coronary artery repair	1 (6.3%)
Aortic valve repair	15 (93.8%)

### Mortality and morbidity

One patient died after enterotomy because of visceral malperfusion after discharge (6.3%). Paraplegia occurred in one patient (6.3%) on admission. One patient with multiorgan failure, lower body malperfusion and stroke recovered after discharge, with the sequelae of impaired intellectual performance. The coronary artery was repaired in one patient (6.3%) with a type A coronary dissection [11].

### Follow-up

**Fig. 2:**
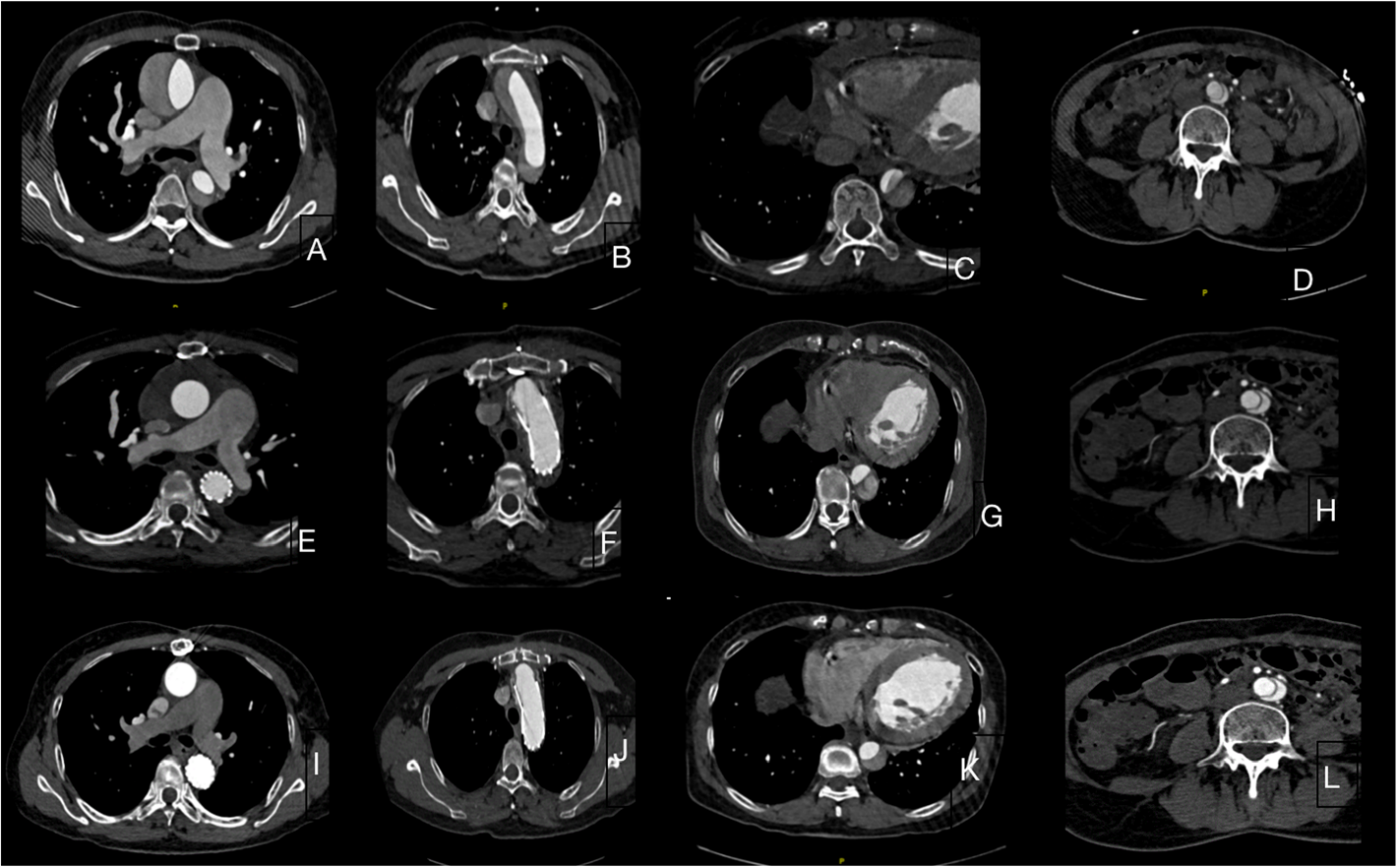
CTA about the aorta at different levels at admission,before discharge, and 3 months after discharge. The upper four pictures stands for the aorta at the bifurcation of the pulmonary artery, aortic arch, and diaphragm and at the bifurcation of the common iliac artery when admitted in hospital (**a**-**d**). The middle and final four pictures stands for the aorta at the bifurcation of the pulmonary artery, aortic arch,and diaphragm and at the bifurcation of the common iliac artery when before discharge(**e**-**h**) and 3 months after discharge(**i**-**l**)

**Fig. 3:**
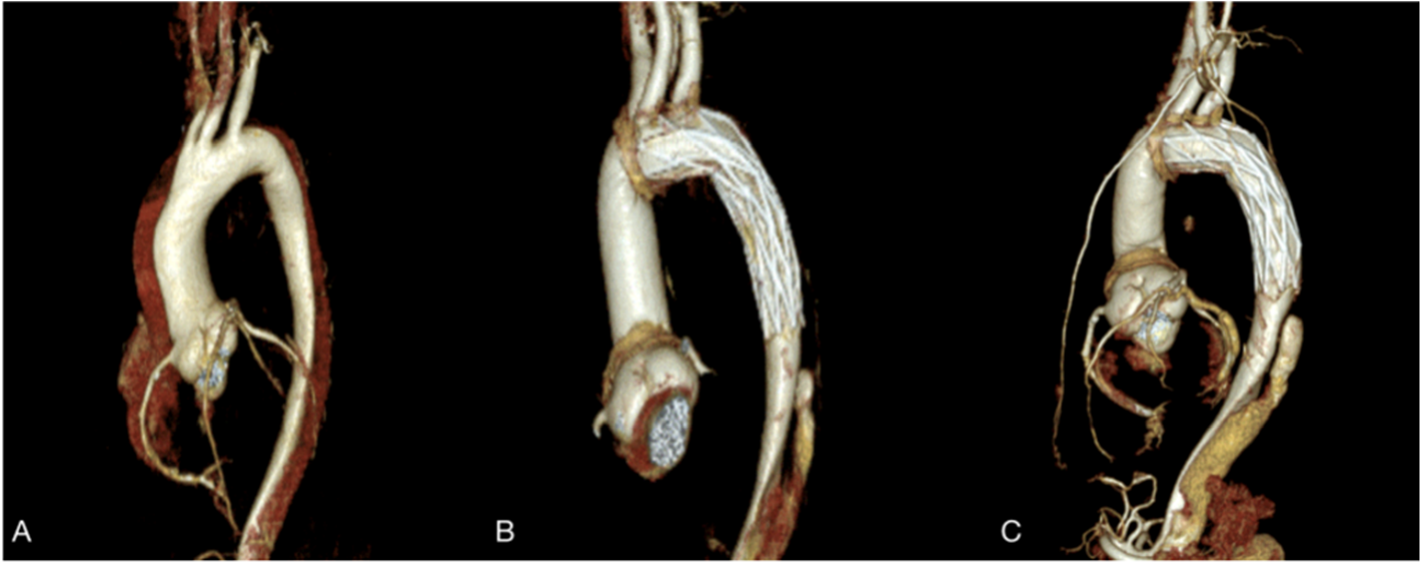
Three-dimensional reconstruction of the aorta at admission, before discharge,and 3 months after discharge(**a**-**c**)

**Fig. 4:**
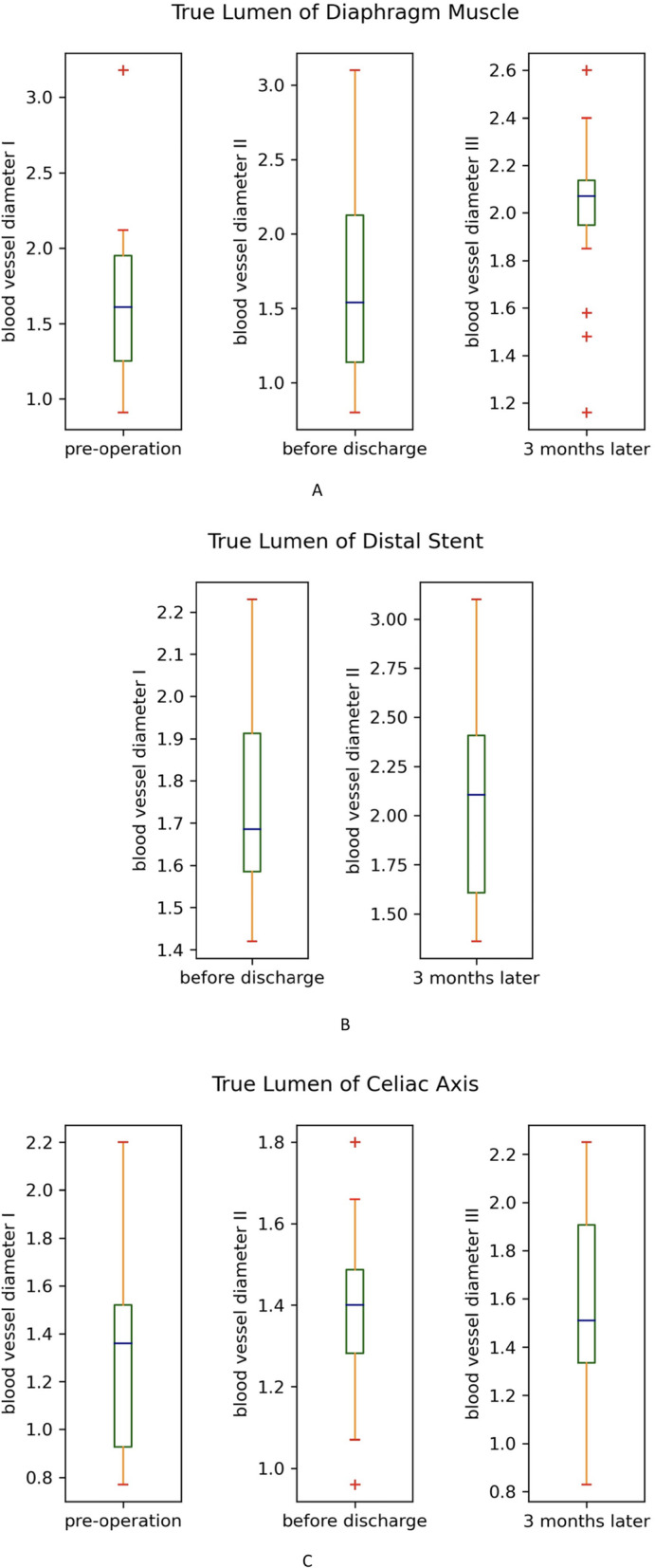
**a**: Changes of true lumen of descending aorta at diaphragm muscle site. **b**: Changes of true lumen of descending aorta at distal stent site. **c**: Changes of true lumen of descending aorta at celiac axis site

Fifteen patients were followed up for 3 months. Changes in the diameter of the ascending aorta, aortic arch, descending aorta at the pulmonary artery bifurcation and diaphragm and at the bifurcation of common iliac arteries are shown in Table 3. The diameter of ascending aorta remained no change because it was replaced with a Dacron tube. The diameter at all other levels were larger before discharge than on admission, except the aortic arch. After 3 months, the diameter of aortic arch and the descending aorta at the level of bifurcation of the pulmonary artery and diaphragm were increased;However, there was little change at the level of bifurcation of common iliac arteries. These changes are shown in Fig. 2 and Table 3. Three-dimensional reconstructions of different level of the aorta are shown in Fig. 3. Changes of true lumen at the distal stent, diaphragm level, and celiac axis of the aorta are shown in Fig. 4.

**Table 3:** Diameter of the aorta with different sites

Site of aorta	Preoperative (mm)	Before discharge (mm)	3 months after discharge	*P*-value
Ascending aorta (mean + SD)	43.0 ± 3.5	28.3 ± 3.1	28.4 ± 2.7	0.14
Aortic arch (mean + SD)	34.4 ± 4.0	30.1 ± 4.1	31.2 ± 3.8	0.07
Descending aorta				
Bifurcation of the pulmonary artery	29.1 ± 3.5	33.5 ± 4.8	34.2 ± 4.2	0.13
Distal stent		31.9 ± 2.6	32.3 ± 3.2	0.01
Diaphragm	29.1 ± 0.4	30.9 ± 0.4	31.2 ± 1.2	0.42
Bifurcation of the common iliac artery	18.5 ± 0.3	20.1 ± 0.3	20.1 ± 0.3	0.66

## Discussion

Acute aortic dissection is a lethal condition that requires emergency surgery to prevent patients from dying of rupture, tamponade and severe aortic valve regurgitation. Additionally, if necessary, surgery is required to correct the dissected aortic root, coronary ostia and distal malperfusion including cerebral and visceral malperfusion. With improvements in surgical techniques, anesthesia and perioperative care, the majority of patients have been salvaged, but still associated with high morbidity and mortality. However, the ideal surgery for acute type A aortic dissection is controversial. Some surgeons suggest ascending aorta replacement with proximal arch replacement and aortic root repair as well. Ando et al. recommended total arch replacement with an elephant trunk to obtain a stronger distal anastomosis and facilitate descending aortic operations [5]. If it is not possible to completely stabilize the dissected membrane or obliterate the false lumen, some surgeons propose total aortic arch replacement with a frozen elephant trunk technique to prevent its later dilatation [4]. The exponentially increasing use of stents for descending aorta, together with encouraging outcomes of the INSTEAD XL trial [12], has incited a prevalent trend toward antegrade stent placement along with replacement of proximal aorta during repair of acute type A aortic dissection [3, 13]. Many studies have reported that aortic arch replacement with the frozen elephant trunk technique can result in excellent aortic remodeling of downstream aorta and can improve long-term outcomes of acute aortic dissection. Some analyses have also found that the frozen elephant trunk technique for aortic arch dissection did not result in unacceptable rates of mortality and morbidity risks [1]. Furthermore, the branched graft technique and the en bloc technique can be used during surgical treatment of aortic arch diseases, including aortic dissection [14]. Additionally, the Y-graft technique and aortic arch debranching are also used by surgeons as aortic therapy [15]. Finally, a prophylactic aortic arch debranching technique has also been reported [4]. However, because of the unclear long-term follow-up outcomes and fewer patients, none of these techniques have emerged as the standard for surgical repair.

In China, total arch replacement using a four-branched graft with implantation of a customized stented graft in the descending aorta (Sun’s procedure) is widely recognized and commended for favorable and safe outcomes and feasibility of reoperation on residual descending aorta [16]. However, Sun’s procedure entails careful dissection, anastomosis of the three brachiocephalic vessels, and time-consuming hemostasis. Thus, the main counter-argument is that greater technical complexity, longer operative time, and extended hypothermic circulatory arrest time may increase patient mortality and morbidity.

To address the limitations of Sun’s procedure and to simplify the procedure, we developed a modified frozen elephant trunk technique for acute Stanford type A dissection, using a fenestrated WeiChuang (Cronus; Microport Medical Co, Ltd., Shanghai, China) stent graft in the arch and circumferentially fixing the stent graft to prevent endoleak. Compared with the most utilized procedures, our technique does not involve dissection of the left subclavian artery, which can be poorly visualized, replacement of all three brachiocephalic vessels, and distal arch end-to-end anastomosis, thereby reducing procedure time. Most importantly, this technique can eliminate tear in the ascending aorta and facilitate false lumen thrombosis and excellent remodeling of the downstream aorta, improving distal malperfusion.

Total arch replacement with frozen elephant trunk technique for acute type A aortic dissection is becoming more accepted in the world, not only because of improved short-term outcome but also better long-term survival. However, it is truly a complex and time-consuming procedure, and unfavorable complications such as paraplegia remain a concern. Our goal for this modified frozen elephant trunk technique is to simplify the procedure with comparable outcomes. The initial result seemed encouraging and effective.

The advantages of this technique are as follows: (1) the frozen elephant trunk is located more proximally, causing limited sacrifice of intercostal arteries and avoiding extensive coverage of the descending aorta. Flores and colleagues demonstrated that extensive coverage of descending aorta with prosthesis represented a strong risk factor for spinal cord ischemia. (2) Judicious dissection and anastomosis of three arch vessels mean less complexity and shorter procedure time. (3) This customized stent graft is convenient for future thoracoabdominal aorta replacement. (4) True lumen is enlarged and offers promising false lumen thrombosis and remodeling. (5) In the initial cases, we found that the margin of the left subclavian artery is generally the source of type I endoleak; thus, we used circumferential anastomosis to prevent endoleak.

Spinal cord ischemia is a serious complication that can occur perioperatively. A length of 10 cm for frozen elephant trunk graft is acceptable, as most cardiac surgeons believe its primary purpose is to stabilize the dissecting membrane and open true lumen expansion downstream [17]. However, Roselli et al. stated that a 10 cm frozen elephant trunk does not offer adequate length to cover the curve of the arch entirely, and a 15-cm device was the most appropriate because 20 cm devices have a tremendous risk of spinal cord injury [13]. In our patients, we advocate 12 cm frozen elephant trunk device, which can greatly decrease the risk of paraplegia as well as provide enough coverage of aortic arch. Selecting a distal landing zone above T7 level seems to decrease the spinal cord ischemia greatly [7]. Pacini et al. claim that spinal cord injury occurs more often in patients with a paten false lumen during follow-up than in those without it [8]. In our series, one patient developed paraplegia before discharge because lower body malperfusion developed soon on admission, and CTA indicated extensive thick thromboses in false lumen, which might be a risk factor for his paraplegia.

According to European guidelines, recommendations for type A intramural hematoma are the same as those for the treating type A aortic dissection [18]. There are 2 patients (12.5%) diagnosed as intramural hematoma presenting with persistent back pain who underwent surgery with our technique. The symptoms completely relieved suggesting satisfactory outcomes for this technique.

Additionally, there have been some reports of several new techniques to treat acute Stanford type A aortic dissection [19, 20]. In contrast to those procedures, we do not use a special tailor-made stent graft, which composed of a 3 cm stent-free vascular graft at the proximal end and a self-expandable stent. The results are comparable.

It is important to note that not all type A aortic dissections are appropriate for this technique. A good candidate for this technique is in the case of a primary tear located in the ascending or descending aorta or lesser curvature of the aortic arch. Other than these locations, the false lumen will be patent, aortic arch will dilate, and type I endoleak will occur.

Using this modified technique, traditional total arch replacement with frozen elephant trunk can be simplified to proximal arch anastomosis, free of careful and complex dissection of three arch vessels and adequate reinforcement of the dissecting aorta. Operation time can be shortened as well as complications minimized. After more than 10 years of dealing with acute type A dissection and more than 500 cases of traditional FET, this modified technique is gradually initiated in selected appropriate cases.

### Limitation

This study was a retrospective study with a small sample size and no control group from a single center. The follow-up period was not long enough after discharge. However, we will continue to adopt this simplified technique with more appropriate patients. Long-term results will be reported later.

## Conclusion

Our modified frozen elephant trunk technique for acute Stanford type A aortic dissection is safe and feasible. Short-term outcomes are encouraging, but long-term outcomes including remodeling of the descending aorta need further investigation.

## Supplementary information

**Additional file 1.** Fenestrated Technique.

## Data Availability

The datasets generated or analyzed during the current study are included in this published article.

## Abbreviations

CPB: Cardiopulmonary bypass
CTA: Computed tomography angiography
FET: Frozen elephant trunk
